# First Bornean orangutan sighting in Usun Apau National Park, Sarawak

**DOI:** 10.3897/BDJ.9.e60753

**Published:** 2021-01-22

**Authors:** Ahmad Ampeng, Jack Liam, Boyd Simpson, Carl Traelholt, Shukor Md Nor, Mohd Shamsul Bahri Abdan-Saleman, Syafiani Osman, Shaffeqe Affendy Zakaria, Badrul Munir Md-Zain

**Affiliations:** 1 Sarawak Forest Department, Petra Jaya, Kuching, Malaysia Sarawak Forest Department, Petra Jaya Kuching Malaysia; 2 Copenhagen Zoo, Department of Research & Conservation, Frederiksberg, Denmark Copenhagen Zoo, Department of Research & Conservation Frederiksberg Denmark; 3 Department of Biological Sciences and Biotechnology, Faculty of Science and Technology, Universiti Kebangsaan Malaysia, Bangi, Selangor, Malaysia Department of Biological Sciences and Biotechnology, Faculty of Science and Technology, Universiti Kebangsaan Malaysia Bangi, Selangor Malaysia

**Keywords:** *Pongo pygmaeus*, orangutan distribution, Usun Apau National Park, Sarawak

## Abstract

Wildlife surveys were conducted in Usun Apau National Park (UANP), Sarawak from Oct 2017 to Oct 2020. This was the first attempt to document fauna diversity in Usun Apau National Park on the UANP plateau at 1200-1400 m a.s.l. On 17 September 2020, 10 AM, we observed an orangutan individual, *Pongo pygmaeus*, over a period of one minute at Libut Camp UANP (E: 114039’.546, N: 2052’36.44) at 1,020 m a.s.l. We also recorded four nests and orangutan vocalisation twice. This observation is important for Bornean orangutan conservation as this was the first orangutan sighting in UANP and well outside the species distribution range for in Sarawak.

## Short communication

Orangutan is the only great ape found in Asia, with two species found on Sumatra, *Pongo abelii* and *Pongo tapanuliensis* (Nater et al. 2017) and one on Borneo (Ancrenaz 2016). Bornean orangutan (*Pongo pygmaeus*) consists of three subspecies, namely, *P. p. pygmaeus* (Sarawak and northwest Borneo), *P. p. wurmbii* (Southwest and central Borneo) and *P. p. morio* (Sabah and northeast Borneo; Goossens 2009, Kamaluddin et al. 2018). All orangutan species are classified as *Critically Endangered* under the IUCN Red List of Threatened Species (Ancrenaz 2016). Most of the remaining orangutan populations are in decline due to logging, poaching, habitat loss and illegal trades (Freund et al. 2016, Hardus et al. 2012).

Orangutan is considered a fully protected species under the Sarawak Wildlife Protection Ordinance 1998. Though they are protected and listed as critically endangered, information relating to their current population size and distribution in Sarawak is still limited with most surveys never having been published (Meredith 1995, Blouch 1997, Pandong et al. 2018). A British team surveyed UANP in the 1950s with no records of orangutan (Arnold 1957). A few other biologists have traversed the area (Stuebing, pers comm 2019) and, in 2012, an international team surveyed UANP for Odonata (Dow 2015), but none of these recorded the occurrence of orangutans.

**Figure 1.**
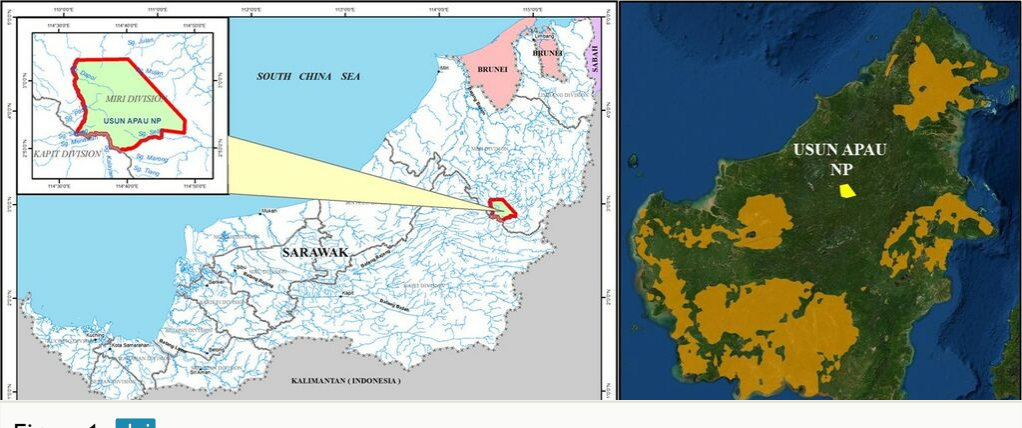
Map of the study area, Usun Apau National Park and Bornean Orangutan distribution.

**Figure 2.**
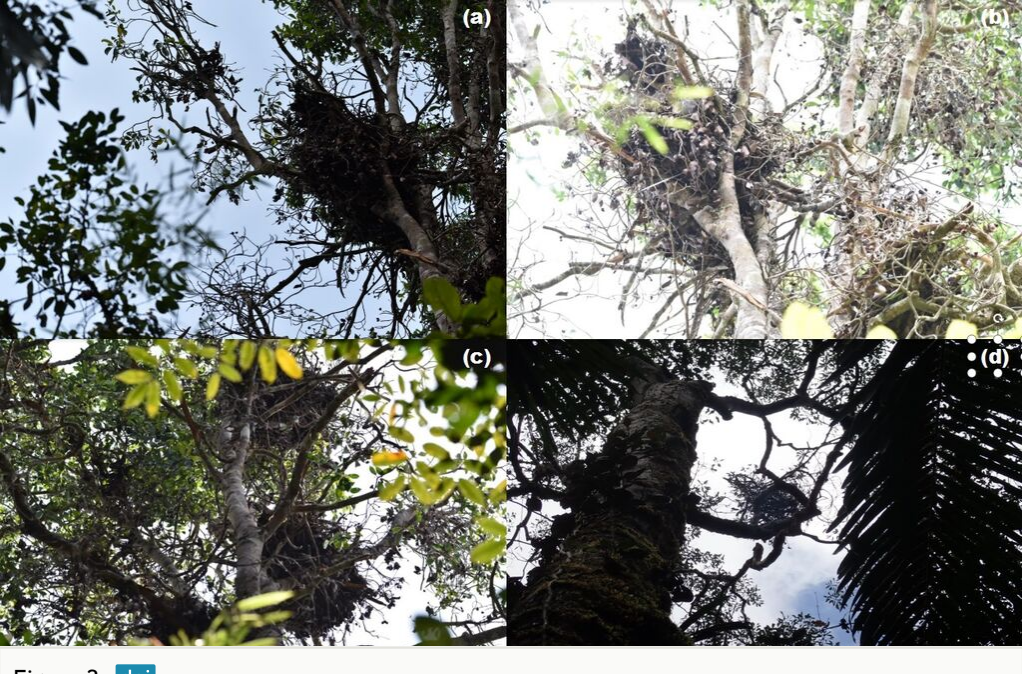
Orangutan nests at Usun Apau National Park: a-c) new nests; d) old nest.

From October 2017 to October 2020, a team led by the Sarawak Forest Department, Universiti Kebangsaan Malaysia and Copenhagen Zoo, Denmark undertook 12 field surveys to Usun Apau National Park (UANP; E: 114 38.258, N: 3 2.252) (Fig. 1). In total, 17 transect lines with a total distance of 73.9 km were set up at an elevation ranging from 1030-1170 m a.s.l. During a routine inspection along an exploratory transect line, one individual of Bornean orangutan was observed on the 17 September 2020 at 10 am near Libut Camp (E: 114039’.546, N: 2052’36.44; 1020 m a.s.l). The sighting lasted about 1 minute. On the same date, four orangutan nests were found in the adjacent area with three new and one old nest (Fig. 2). We also heard orangutan vocalise twice. We undertook additional aerial surveys using a drone (DJI Phantom 4 Pro and DJI Inspire) fitted with FLIR-camera to extend the survey range. FLIR has been successfully used to record moving and nesting wild orangutan at night (Dahlen and Traeholt 2018, Abdul-Mutalib 2019), but we were unable to confirm the presence of additional nests from daytime aerial photographs (photographic analyses are still ongoing) and orangutans from night surveys using FLIR.

It is estimated that at least 104,700 *P. pygmaeus* still exist on Borneo (Ancrenaz 2016). In Sarawak, orangutan can be found in Batang Ai National Park, Ulu Sebuyau National Park, Sedilu National Park and Lanjak Entimau Wild Life Sanctuary which are all totally protected areas (Ampeng et al. 2016, Tisen and Silang 2016, Pandong et al. 2018). Based on distribution map by Wich et al. (2008) and Ancrenaz (2016), the orangutan sighting in UANP constitutes a new orangutan record outside its published habitat range in Sarawak. Since UANP is located at the assumed distribution borderline between *P. p. pygmaeus* and *P. p. morio* (Wich et al. 2008), it is unclear to which subspecies the individual observed in UANP belongs. Although male orangutans are known to travel long distances between various areas, UANP is 2-300 km distant from orangutan’s current known distribution range. Therefore, any individuals recorded in UANP are likely not only to be a lone male traversing into a different area.

In the near future, the survey team will continue to visit UANP and adjacent areas to obtain more information of a potential small isolated orangutan population in Sarawak. In addition, where possible, molecular phylogenetic analyses need to be carried out to clarify its genetic origin and belonging (Kamaluddin et al. 2018, Abdul-Manan et al. 2020) and provide a clear and robust taxanomic distinction (Goossens 2009, Zainudin et al. 2010). Our findings suggest that the Sarawak orangutan distribution range must be updated along with relevant conservation implications.

## Hosting institution

Sarawak Forest Department
